# Second-Generation Cephalosporins-Associated Drug-Induced Liver Disease: A Study in VigiBase with a Focus on the Elderly

**DOI:** 10.3390/ph14050441

**Published:** 2021-05-07

**Authors:** Mariana Sipos, Andreea Farcas, Daniel Corneliu Leucuta, Camelia Bucsa, Madalina Huruba, Cristina Mogosan

**Affiliations:** 1 Department of Pharmacology, Physiology and Physiopathology, Faculty of Pharmacy, “Iuliu Haţieganu” University of Medicine and Pharmacy, 400349 Cluj-Napoca, Romania; mariana.sipos89@gmail.com (M.S.); huruba.madalina@umfcluj.ro (M.H.); cmogosan@umfcluj.ro (C.M.); 2 Drug Information Research Center, “Iuliu Hatieganu” University of Medicine and Pharmacy, 400349 Cluj-Napoca, Romania; cim@umfcluj.ro; 3 Department of Medical Informatics and Biostatistics, “Iuliu Hatieganu” University of Medicine and Pharmacy, 400349 Cluj-Napoca, Romania; dleucuta@umfcluj.ro

**Keywords:** cephalosporins, VigiBase, adverse drug reaction, hepatotoxicity

## Abstract

Background: The objective of this study was to characterize individual case safety reports (ICSRs) and adverse drug reactions (ADRs) related to second-generation cephalosporins and resulting in hepatobiliary disorders, in VigiBase, WHO global database. Methods: All second-generation cephalosporins hepatobiliary ADRs reported up to July 2019 were included. Characteristic of cephalosporins and ADRs, aside from disproportionality data were evaluated. Results: A total of 1343 ICSRs containing 1585 ADRs were analyzed. Cefuroxime was suspected to have caused hepatobiliary disorders in most cases—in 38% of adults and in 35% of elderly. Abnormal hepatic function was the most frequent ADR, followed by jaundice and hepatitis. For 49% of the ADRs reported in the elderly and 51% in the adult population, the outcome was favorable, with fatal outcome for 2% of the adults and 10% of the elderly. Higher proportional reporting ration (PRR) values were reported in the elderly for cefotetan-associated jaundice, cefuroxime-associated acute hepatitis and hepatitis cholestatic as well as for cefotiam and cefmetazole-associated liver disorder. Conclusion: Hepatobiliary ADRs were reported for 2nd generation cephalosporins, with over 50% of cases in adults, without gender differences. Cholestatic hepatitis was predominately reported in the elderly and this category was more prone to specific hepatic reactions.

## 1. Introduction

Drug-induced liver injury (DILI) is a major cause of morbidity and mortality and is considered the principal cause of market drug withdrawal [1]. Although the frequency of serious antibiotic-induced hepatotoxicity is low compared to the yearly exposure to antibiotics, drug-induced hepatotoxicity remains one of the reasons for antibiotic withdrawal after product launch [2,3,4]. The pathogenesis behind idiosyncratic DILI is not well understood, but is generally thought to be unpredictable and may have serious consequences [3]. Antibiotic-induced hepatotoxicity is usually asymptomatic and transient; nonetheless, in rare cases, significant morbidity and the need for liver transplantation and death from acute liver failure have been reported [2].

Cephalosporins are a class of antibiotics frequently prescribed, grouped into five generations based on their spectrum [5,6,7]. Second generation cephalosporins have a relatively broad spectrum activity, a variety of methods of administration and are often prescribed to treat respiratory infections [6,8,9], urinary tract infections, soft tissue infections and for surgical prophylaxis [5,8,9,10]. Cephalosporins prescribed by age groups showed higher prescribing rates for the adult population (18–64 years old) (n = 32,828), followed by those under 18 years old (n = 15,169), while the elderly population had lower prescribing rates (n = 7016) [7]. Although stewardship programs were implemented to reduce cephalosporins use and have managed to reduce the consumption where applied [11,12], an overall increase in consumption was reported globally [13,14]. Cephalosporins are considered to have low toxicity and are generally safe [5,10,15]. They are only rarely associated with severe hepatotoxic reactions with clinical symptoms, which are usually reversed after drug withdrawal [8]. The cholestatic mechanism is most often described for liver toxicity associated with the use of cephalosporins. Mild cholestasis with a portal, lobular mixed inflammation and focal bile duct injury are usually observed. The symptoms of liver toxicity can manifest within a few days of treatment initiation [2,16], but can also have a later onset [17]. Another hepatic adverse reaction described for cephalosporins is the formation of biliary sludge due to precipitation of the different salts, reversible following the drug’s withdrawal as the crystals solubilize [18].

It has been suggested that there are more factors that can favor DILI. Age has been cited as a risk factor, but the at-risk age groups and DILI mechanism differ among published evidence [3,19,20]. A new safety signal was recently detected for ceftriaxone-induced hepatitis in elderly patients (≥75 years old) in VigiBase, the unique World Health Organization (WHO) global database of individual case safety reports (ICSRs) [21]. Female patients and patients with a history of chronic liver disease, HIV and obesity were also suggested to be at risk for developing DILI more often [20].

There have been few published case reports of liver disease associated with second-generation cephalosporins [15,22,23,24]. The objective of this study was to characterize ICSRs and adverse drug reactions (ADRs) reported for the second-generation cephalosporins and resulting in *hepatobiliary disorders* in VigiBase, in the light of the reported increased consumption of cephalosporins [13,14,25] and the numerous studies pointing out to antibiotic-induced liver disease [17,26,27,28,29,30,31,32]. The study also aims to explore the association between hepatobiliary disorders and second-generation cephalosporins by the means of disproportionate reporting stratified by age group and sex.

## 2. Results

### 2.1. Characteristics of Case Reports

Up until 1 July 2019, 1343 case reports that included second-generation cephalosporins as *suspect*/*interacting* for adverse reactions within the *hepatobiliary disorders System Organ Class* (*SOC*) were reported in VigiBase. Over 50% of all case reports were for adult patients (18–64 years old) and approximatively 28% were reported for the elderly. The reports were evenly distributed between males and females for both age categories mentioned above (Table 1).

Health-care professionals reported most cases in both age groups, with 59.34% of cases reported for the adult population and 55.85% for the elderly.

Out of the 1343 cases, 1336 have had a second-generation cephalosporin listed as *suspect*, while in 7 cases, cephalosporins were listed as *interacting* in causing hepatobiliary ADRs. Among the cases with cephalosporin suspected for the reaction, in 1278 reports, one cephalosporin was suspected; in 50 reports, 2 concomitant cephalosporins were suspected, and in 8 cases, there were 3 suspected cephalosporins. Based on the selected criteria, the following drugs were mentioned as suspect co-medication: paracetamol (47 cases), amoxicillin/clavulanic acid (31 cases), valproic acid, atorvastatin and erythromycin (each reported in 9 cases), simvastatin (7), carbamazepine (6), isoniazid (3) and rifampicin in 2 cases. Cotrimoxazole was not mentioned as suspect in our cohort.

Cefuroxime was suspected to have caused hepatobiliary disorders in most cases reported in both age groups (38% of the cases in adult population and 35% in the elderly population), followed by cefaclor (15%) for the adult population and cefotiam (11%) for the elderly (Table 1).

**Figure 1:**
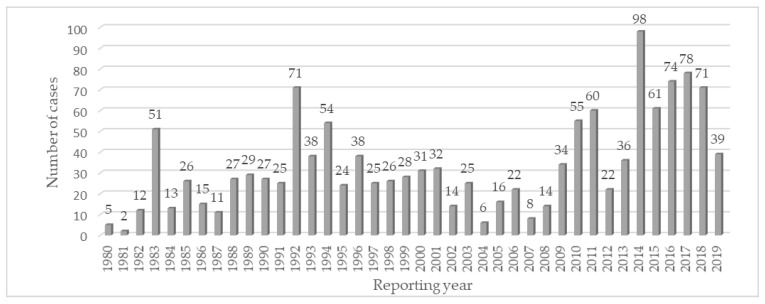
Time trend of individual case safety report in the Hepatobiliary System Organ Class cases for *second-generation cephalosporins*.

Over 75% of the cases reported for cephalosporins over the years have come from spontaneous reports. Although not constant, there was a general increase in the trend of reporting from 1980 to 2018 with several peaks, the highest one being in 2014 (Figure 1).

### 2.2. Characteristics of ADRs

*Hepatic function abnormal* was the ADR most frequently reported, followed by *jaundice* and *hepatitis* in both adult and elderly populations (Table 2), with one or more ADRs reported per patient. *Hepatocellular injury* was more frequently reported in adult patients (5%) compared to elderly patients (3%), while *cholestatic hepatitis* was more frequently reported in the elderly (8%) as compared to adults (4%).

For almost half of the reported ADRs in both age groups (49% for the elderly and 51% for adult), the outcome was favorable (*recovered* or *recovering* from the ADR). A fatal outcome where the hepatobiliary reaction may have been contributory was reported for 4 patients in the adult group and in 8 patients in the elderly group.

The time to onset of the ADR was less than 7 days in most cases (36% in the adult population and 38% in the elderly), followed by 8–14 days (Table 2).

For over 30% of ADRs, the drug was withdrawn in all age groups, which led to the ADR being abated in more than 75% of the overall population, with a slightly better dechallenge outcome for the adult population in which the reaction abated in 80%, as compared to almost 72% in the elderly.

### 2.3. Disproportionality Analysis

For the analysis of disproportionality data, the cohort was stratified by three main age groups as provided by the UMC (18–44, 45–64 and ≥65 years old) and by sex. All hepatobiliary ADRs associated with cephalosporins presenting disproportionality in the overall and elderly groups are listed in Table 3, stratified by age groups, and the ones presenting disproportionality in the elderly, stratified by gender, are presented in Table 4.

Disproportionality data (Table 3) showed that there was a stronger drug-ADR association for the elderly as compared to other adult population categories for associations such as *cefotetan*-associated *jaundice* and *cefuroxime*-associated *acute hepatitis*. The same was reported for *cefuroxime*-associated *hepatitis cholestatic*, and for *cefotiam* and *cefmetazole*- associated *liver disorder*.

Out of the total 49 reported ADRs with disproportionality in the elderly group, in 30 ICSRs, the cephalosporin was the only drug considered as suspect, while for the other 19 cases, between 2 and 6 drugs known to cause hepatobiliary disorders were considered as suspect for the ADR. Besides cephalosporins, suspected drugs in the aforementioned cases were: amoxicillin + clavulanic acid in 4 cases, paracetamol in 2 and carbamazepine. Other 8 drugs labeled as suspect were mentioned: ceftriaxone, azithromycin, cilastatin, fluoroquinolones (moxifloxacin, ciprofloxacin, ofloxacin) or fibrates (fenofibrate, ciprofibrate) were mentioned once each.

When looking at the disproportionality data stratified by gender for the ADRs reported for the elderly, there were certain differences as presented in Table 4. *Cefuroxime- associated cholestatic hepatitis* had a greater PRR value for male elderly as compared to female elderly patients.

## 3. Discussion

In this study, we investigated hepatobiliary disorders related to second-generation cephalosporins, still largely used in clinical practice. To the best of our knowledge, this is the first research conducted in VigiBase on liver disorders associated with second-generation cephalosporins, presenting data stratified by age groups and having a focus on the elderly. A research conducted in VigiBase that has detected a new signal of hepatitis for ceftriaxone (a third-generation cephalosporin) in patients of 75 years and older [21], aside from the numerous studies pointing out to antibiotic induced liver disease [17,26,27,28,29,30,31,32] and the increased consumption of cephalosporins [13,14], laid the ground for the need of further assessment of hepatic adverse events associated with the use of cephalosporins.

In the global VigiBase data set, the age difference for reports of hepatobiliary disorders associated with the use of cephalosporins was evident. Hepatobiliary disorders were reported predominately for the adult population (50.9%), followed by the elderly (27.6%), consistent with the age distribution reported by Hunt et al. when assessing 236 drugs associated with hepatotoxicity in VigiBase, where almost 65% events were reported for the adult population and 32% for the elderly [30]. However, in the Hunt et al. study, the elderly exhibited an increased liver event reporting, compared to the other age groups, and for 10 drugs, mainly antibiotics, including amoxicillin/clavulanate and flucloxacillin [30]. Age over 65 [2] and even over 55 [28] has been reported as a risk factor for liver events. Similarly, we too observed in the disproportionality data that age is a risk factor for the development of certain liver events such as acute hepatitis and cholestatic hepatitis due to cefuroxime.

Our study shows that cases for hepatobiliary disorders associated with the use of second-generation cephalosporins are almost similarly distributed between men and women, both in the overall cohort, and in the elderly. The DILI studies on Chinese population showed a predominance in men [26,27], while a study conducted in a Spanish DILI registry found no gender differences [17]. In contrast, female predominance was reported in other studies [28,33,34].

When analyzing ADRs’ reporting trends over time, the following factors need to be taken into consideration: global antibiotic use [13,14], antibiotic resistance and stewardship programs targeting to reduce prescribing of all cephalosporin molecules [12], as well as factors (such as age or drug-specific factors) that might be influencing data reporting in VigiBase [30]. Although some studies have reported a decrease in cephalosporin prescribing in Germany after 2011 [12] or between 2012 and 2017 [11], global use of cephalosporins is reported to have an ascending trend [13,14]. Therefore, we might also suspect that the increase in ADRs reporting from the present analysis follows large utilization of cephalosporins [7,35].

Case reports for hepatobiliary disorders associated with the use of second-generation cephalosporins originated mainly from Asia, followed by Europe and America. This could be explained by the fact that DILI incidence reported in the Asian population is much higher compared to western Europe [36], plus the increased cephalosporins use in the Asian population [25,26] and the fact that antibiotic use is being reported as the third cause of DILI in the Chinese population [27]. Reports in VigiBase originate mainly from these three continents, while for other regions (Africa, Oceania), data on hepatic disorders is scattered as the pharmacovigilance systems are usually less developed.

The large availability of cefuroxime for oral and injectable use [9], along with increased prescribing [14], explain the increased number of case reports of ADRs in VigiBase for this drug, compared to the other second-generation cephalosporins.

Cholestatic hepatitis was predominately reported for the elderly (8%) compared to the adult population (4%), results consistent with what other studies have reported [19,29,30], emphasizing that cholestatic mechanism for the liver injury is more frequent with older age. Disproportionality data support the existence of a stronger association between cefuroxime and cholestatic hepatitis in the elderly (Proportional Reporting Ratio (PRR) 2.52, 95% CI 1.13, 3.65) compared to the adult population aged 45–64 years old (PRR 1.57 95% CI 1.57, 0.89), while no association was found for the 18–44 age group, thus suggesting that cholestatic hepatitis associated with the use of cefuroxime is more likely to happen in the elderly.

Disproportionality data for the elderly population by sex, suggest that hepatitis cholestatic associated with the use of cefuroxime was more likely to appear in male patients as opposed to female patients (PRR 2.72 95% CI 1.55, 4.27 vs. PRR 1.38 95% CI 0.62, 2.00). Other studies have reported a male predominance for cholestatic injuries in their cohort [19], thus supporting our finding. Jaundice was strongly associated with the use of cefotetan in elder females, while for cefaclor, the predominance was in males. Some studies showed a greater incidence of jaundice in females [26] and supported the hypothesis that the female gender is a risk factor.

The fatal outcome in our data was reported in 4% of cases in the overall cohort. However, the ADR was considered contributory in 1% of cases. Considering that our data comes from spontaneous reporting, the information on to what extent the hepatobiliary ADRs might have contributed to death is not always available. The results of a study conducted in a Spanish registry reported a 2% fatal outcome for patients with mixed liver injury and an average of 52 years old, 5% for cholestatic liver injury and an average age of 61 years old and 7% for hepatocellular liver injury in patients with an average age of 51 years old [17]. A much higher fatal outcome was found for the elderly in our study (10%), as older patients are generally prone to a more negative outcome in case of ADRs.

Time to onset analysis showed that hepatobiliary disorders had an early onset in over 46% of cases, while late-onset (of over 15 days) was reported in 10% of cases in the overall cohort. No important differences have been reported for different age categories. Case reports have showed the debut of ADRs associated with the use of cefuroxime ranging from 4 days [24] to 11 days [2]. Nonetheless, other studies have reported a later onset of hepatobiliary disorders associated with the use of antibiotics. Andrade et al. have reported a mean time to onset of 53 days (in a range of 30–76 days) for mixed liver injury and 119 days (range 47–192) for hepatocellular liver injury in a study analyzing liver disease cases that were suspicious of being related to drugs or toxins [17]. An early onset of the drug reaction is usually more rapidly recognized, gives the opportunity of a drug withdrawal, and usually is related to a positive outcome for the ADR, once the drug was withdrawn [20]. In our case, for 31% of ADRs, the drug was withdrawn, with a slightly greater percentage for the elderly (34%). This action has led to the ADR being abated in over 80% percent of ADRs for the adult population, with a slightly lower percentage (72%) for the elderly.

### 3.1. Limitations

Some limitations of this study must be outlined. For the ADRs’ assessment, we only selected the hepatobiliary disorders where the cephalosporin was the suspect drug, excluding the case reports where the cephalosporin was mentioned as interaction. Nonetheless, our aim was to assess the ADRs for suspected cephalosporins, excluding the cases where the DILI might have had another cause.

As data comes from spontaneous reporting, under-reporting of ADRs can be considered as a limitation. In addition, the fact that the information comes from a variety of sources, and the probability that the suspected adverse effect is drug-related is not the same in all cases and can be considered a limitation as well. Another limitation in this aspect was the limited information regarding dechallenge actions and little or no data for rechallenge actions. Moreover, we did not consider drug-drug interactions or other potential factors that could modify hepatotoxicity in the reported case. Hepatotoxicity is likely to be influenced by various other factors (e.g., food supplements, comedication, comorbidities) that can influence the development, magnitude and outcome of the ADRs, which were not or could not be evaluated given the available data. Causality between adverse reaction and drug can vary from report to report, dependent on reporting and collecting of data.

### 3.2. Strengths

The strength of our study lies in the use of the largest databases of spontaneous reports and the extraction of all the data available for ADRs in the hepatobiliary SOC associated with the use of second-generation cephalosporins. This provided us with the opportunity to study disproportionality data of second-generation cephalosporins and allowed us to stratify the data by age and sex, studying risk factors associated with drug-induced hepatic disorders.

## 4. Materials and Methods

### 4.1. Data Source and Analysis

VigiBase, a database maintained by the Uppsala Monitoring Centre (UMC), was used as the data source for this study. VigiBase includes case reports from more than 130 countries representing over 90% of the world’s population and contained (up to the date of the data extraction) more than 19 million ICSRs submitted by national pharmacovigilance centers since 1967 [37]. Adverse drug reactions in VigiBase are classified according to the hierarchical structures of the Medical Dictionary for Regulatory Activities’ (MedDRA) latest version of the time of reporting. All ICSRs reported up to July 1st 2019 for second-generation cephalosporines (ATC code J01DC) and containing reactions (as Preferred Terms—PTs) within the hepatobiliary disorders SOC were received from UMC deduplicated. All ICSR with a second-generation cephalosporin (referred to as cephalosporin(s)) as the suspect or interacting drug were analyzed in this study. Fixed combinations were excluded from the analysis. Concomitant drugs (basis suspect) with possible hepatotoxic potential, were extracted according to a ranking of the 10 most frequently reported drugs associated with liver injury from a previous study in VigiBase: amoxicillin/clavulanic acid, atorvastatin, carbamazepine, cotrimoxazole, erythromycin, isoniazid, paracetamol, rifampicin, simvastatin and valproic acid [31].

Descriptive statistics were used to characterize the case reports extracted from VigiBase. All reports were analyzed for sex, age, reporting region, report type and reporter, serious and seriousness criteria, cephalosporin(s) and ADRs. One report could include more than one ADR and more than one suspected or interacting drug. All ADRs with at least one suspected cephalosporin reported were characterized for outcome, time to onset, reaction duration and outcome of dechallenge/rechallenge action, if performed. All data is presented for the overall dataset and stratified by age (18–64 years—adult population and ≥65 years—elderly population).

### 4.2. Disproportionality Analysis

The disproportionality data was provided by the UMC stratified by age group (0–17, 18–44, 45–64 and ≥65 years old) and sex. Disproportionality analysis is the best approach to signal detection in pharmacovigilance [38,39]. The strength of drug-ADR dependency was defined by PRR, meaning the proportion of the event of interest (in our case hepatobiliary disorders) for a specific drug (in our case cephalosporins), compared to the proportion of the event of interest for all other drugs from the database [39,40,41]. Proportional Reporting Ratio values, with their 95% confidence intervals (CIs), were used as provided by the UMC. A lower value of the 95% confidence interval of the PRR >1 associated with ≥5 cases was considered a positive drug-ADR association [41,42].

For the disproportionality analysis in the current study, we have selected the MedDRA PTs that suggest a liver injury, according to the Council for International Organizations of Medical Sciences (CIOMS) Reporting Adverse Drug Reactions Definitions of Terms and Criteria for their Use [43]. The following terms were therefore included in the present analysis: *acute hepatic failure, cholangitis (all types), hepatitis (all types), hepatocellular injury, jaundice (all types), liver disorder and liver injury (all types)*.

## 5. Conclusions

The results from our study complement data available in literature emphasizing the connection between the use of cephalosporins and hepatobiliary ADRs and a higher risk for some of these ADRs with age. Our study revealed that cholestatic hepatitis was predominately reported for the elderly, emphasizing that cholestatic mechanism for liver injury is more frequent with older age. This aspect was supported by the disproportionality data showing that cholestatic hepatitis associated with the use of cefuroxime is more likely to happen in the elderly. Generally, hepatobiliary disorders were reported predominately for the adult population, and were similarly distributed between men and women, both in the overall cohort, as well as in the elderly.

## Figures and Tables

**Table 1:** Characteristics of the individual case safety reports, overall and by age categories.

Characteristics	Overall ^a^		18–64 Years Old		≥65 Years Old	
	N = 1343		N = 684		N = 371	
**Gender**	**N**	**%**	**N**	**%**	**N**	**%**
*Female*	624	46.46	330	48.25	184	49.60
*Male*	632	47.06	350	51.17	182	49.06
*Not known*	87	6.48	4	0.58	5	1.35
**Region**	**N**	**%**	**N**	**%**	**N**	**%**
*Asia*	452	33.66	264	38.60	140	37.74
*Europe*	427	31.79	214	31.29	135	36.39
*Americas*	382	28.44	168	24.56	70	18.87
*Oceania*	78	5.81	35	5.11	26	7.01
*Africa*	4	0.3	3	0.44	0	0
**Report Type**	**N**	**%**	**N**	**%**	**N**	**%**
*Spontaneous*	1019	75.87	491	71.78	277	74.66
*Report from study*	48	3.57	23	3.36	16	4.31
*Post Marketing Surveillance/Special monitoring*	46	3.43	29	4.24	13	3.5
*Not known*	230	17.13	141	20.62	65	17.52
**Notifier ^b^**	**N = 1401**	**%**	**N = 718**	**%**	**N = 391**	**%**
*Health Care Professionals ^c^*	758	54.10	401	55.85	232	59.34
*Non-Health Care Professionals ^d^*	72	5.14	39	5.43	20	5.12
*Not known*	571	40.76	278	38.72	139	35.55
**Seriousness ^e^**	**N = 1416**	**%**	**N = 717**	**%**	**N = 396**	**%**
*Caused/Prolonged Hospitalization*	186	13.14	92	12.83	58	14.65
*Life threatening*	32	2.26	14	1.95	10	2.53
*Death*	30	2.12	11	1.53	16	4.04
*Disabling/Incapacitating*	7	0.49	3	0.42	4	1.01
*Congenital anomaly/Birth defect*	1	0.07	0	0	0	0
*Not known*	1160	81.92	597	83.26	308	77.78
**Serious**	**N**	**%**	**N**	**%**	**N**	**%**
*Yes*	491	36.56	255	37.28	147	39.62
*No*	137	10.2	80	117	48	12.94
*Not known*	715	53.24	349	51.02	176	47.44
**Second-generation cephalosporins ^f^**	**N**	**%**	**N**	**%**	**N**	**%**
*Cefuroxime*	480	35.74	260	38.01	130	35.04
*Cefaclor*	220	16.38	100	14.62	32	8.63
*Cefoxitin*	110	8.19	66	9.65	26	7.01
*Cefotiam*	99	7.37	48	7.02	39	10.51
*Cefotetan*	89	6.63	50	7.31	29	7.82
*Cefmetazole*	81	6.03	40	5.85	34	9.16
*Cefamandole*	81	6.03	37	5.41	24	6.47

^a^ Regardless of age; ^b^ More than one notifier per report was possible; ^c^ This includes physicians, pharmacists and other healthcare professionals; ^d^ This includes consumers/non-healthcare professionals and lawyers; ^e^ More than one seriousness criteria per report was possible; ^f^ Other second-generation cephalosporins were present in less than 5% of cases.

**Table 2:** Most frequent adverse drug reactions and outcomes of adverse drug reactions in the Hepatobiliary System Organ Class.

Characteristics	Overall ^a^		18–64		≥65	
	N = 1585		N = 813		N = 429	
**ADR**	**N**	**%**	**N**	**%**	**N**	**%**
*Hepatic function abnormal*	512	32.30%	279	34.32%	138	32.17%
*Jaundice*	236	14.89%	115	14.15%	64	14.92%
*Hepatitis*	166	10.47%	93	11.44%	36	8.39%
*Hyperbilirubinaemia*	112	7.07%	51	6.27%	33	7.69%
*Hepatitis cholestatic*	94	5.93%	35	4.31%	33	7.69%
*Hepatocellular injury*	68	4.29%	43	5.29%	14	3.26%
*Liver disorder*	58	3.66%	32	3.94%	22	5.13%
*Drug-induced liver injury*	45	2.84%	25	3.08%	8	1.86%
*Hepatic failure*	35	2.21%	16	1.97%	10	2.33%
*Cholestasis*	33	2.08%	12	1.48%	12	2.80%
**Outcome**						
*Recovered/resolved*	513	32.37%	286	35.18%	140	32.63%
*Recovering/resolving*	232	14.64%	128	15.74%	69	16.08%
*Not recovered/not resolved*	181	11.42%	101	12.42%	56	13.05%
*Fatal*	71	4.48%	20	2.46%	41	9.56%
*Recovered/resolved with sequelae*	16	1.01%	9	1.11%	2	0.47%
*Died–reaction may be contributory*	15	0.95%	5	0.62%	10	2.33%
*Died–unrelated to reaction*	12	0.76%	4	0.49%	8	1.86%
*Unknown*	660	41.64%	260	31.98%	103	24.01%
**Time to onset ^b^**						
*1–7 days*	547	34.51%	293	36.04%	164	38.23%
*8–14 days*	185	11.67%	111	13.65%	52	12.12%
*15–29 days*	80	5.05%	46	5.66%	26	6.06%
*≥30 days*	71	4.48%	42	5.17%	22	5.13%
*Unknown*	702	44.29%	321	39.48%	165	38.46%
**Dechallenge Action—Drug withdrawn**	**493**	**31.1%**	**245**	**30.14%**	**146**	**34.03%**
**Dechallenge outcome**						
*Reaction abated*	385	78.09%	198	80.82%	105	71.92%
*No effect observed*	66	13.39%	29	11.84%	22	15.07%
*Effect unknown*	34	6.90%	17	6.94%	12	8.22%
*Fatal*	6	1.22%	0	0.00%	6	4.11%
*Missing data*	2	0.41%	1	0.41%	1	0.68%

^a^ Regardless of age; ^b^ Data on time to onset was reported for 56% of ADRs in the dataset analyzed; ADRs (Adverse Drug Reactions) were reported using the MedDRA Preferred Term (PT); MedDRA—Medical Dictionary for Regulatory Activities.

**Table 3:** Disproportionality for hepatobiliary adverse drug reactions associated with the use of cephalosporins by age groups.

Cephalosporin	ADRs ^a^	Overall ^b^		≥65 Years Old		18–44 Years Old		45–64 Years Old	
		N	PRR (95% CI)	N	PRR (95% CI)	N	PRR (95% CI)	N	PRR (95% CI)
Cefotetan	Jaundice	37	2.73 (1.98, 4.71)	10	3.15 (1.70, 4.86)	14	2.10 (1.25, 3.35)	7	**1.39 (0.66, 2.05)**
Cefotiam	Liver disorder	16	2.83 (1.74, 4.57)	10	**6.96 (3.75, 10.71)**	2	1.66 (0.41, 2.07)	4	1.86 (0.7, 2.56)
Cefuroxime	Hepatitis acute	9	1.02 (0.53, 1.55)	6	**2.52 (1.13, 3.65)**	2	0.57 (0.14, 0.71)	1	0.36 (0.05, 0.41)
Cefuroxime	Hepatitis cholestatic	48	2.03 (1.53, 3.55)	18	**2.04 (1.28, 3.32)**	5	0.72 (0.30, 1.02)	12	1.57 (0.89, 2.46)
Cefmetazole	Liver disorder	12	3.20 (1.82, 5.01)	5	5.58 (2.33, 7.91)	2	**2.97 (0.74, 3.71)**	5	4.17 (1.74, 5.90)

^a^ ADRs (adverse drug reactions) were reported using the MedDRA Preferred Term (PT); ^b^ Regardless of age; N—number of ADRs; PRR—Proportional Reporting Ratio; CI—Confidence Interval; bold represents non overlapping Cis. A lower value of the 95% confidence interval of the PRR >1 associated with ≥5 cases was considered a positive drug-ADR association.

**Table 4:** Disproportionality for hepatobiliary adverse drug reactions associated with the use of cephalosporin by gender in the elderly.

Cephalosporin	ADRs ^a^	≥65 Years Old ^b^		Female		Male	
		N	PRR (95% CI)	N	PRR (95% CI)	N	PRR (95% CI)
Cefotetan	Jaundice	10	3.15 (1.70, 4.86)	9	**6.11 (3.19, 9.30)**	1	0.58 (0.08, 0.66)
Cefotiam	Liver disorder	10	6.96 (3.75, 10.71)	7	**9.84 (4.71, 14.55)**	2	2.77 (0.69, 3.46)
Cefaclor	Jaundice	9	1.34 (0.70, 2.04)	2	0.56 (0.14, 0.70)	7	**2.39 (1.14, 3.53)**
Cefuroxime	Hepatitis cholestatic	18	2.04 (1.28, 3.32)	6	1.38 (0.62, 2.00)	12	**2.72 (1.55, 4.27)**

^a^ ADRs (adverse drug reactions) were reported using the MedDRA Preferred Term (PT); ^b^ Gender of patients include male, female and not known; N—Number of ADRs; PRR—Proportional Reporting Ratio; CI—Confidence Interval; bold represents non overlapping CIs with higher PRR. A lower value of the 95% confidence interval of the PRR >1 associated with ≥5 cases was considered a positive drug-ADR association.

## Data Availability

Data sharing not applicable.
